# Sir Nicholas Gilbourne's (magical) cross-over trial of 1631

**DOI:** 10.1177/09677720241304738

**Published:** 2024-12-15

**Authors:** Max Cooper, Sarah Cooper

**Affiliations:** 1 Department of Primary Care and Public Health, 12190 Brighton and Sussex Medical School, Brighton, UK; 2 Department of Neurology, 156800 Royal Sussex County Hospital, Brighton, UK

**Keywords:** Seventeenth century, cross-over trials, *n*=1 studies, controlled trials, fair comparison, weapon salve, magic

## Abstract

We describe a basic ‘cross-over’ trial undertaken by Sir Nicholas Gilbourne of Kent, England, in or before 1631. This was used to test the effectiveness of ‘weapon salve’, an ointment claimed to cure ‘sympathetically’ (i.e. remotely) by application to the weapon that inflicted an injury. Gilbourne reports very basic outcomes but these represent key stages of a modern cross-over trial: no treatment, treatment, no treatment, treatment. We discuss the value of such historical vignettes – even a magical one – for medical students in two respects: understanding research methodology and learning about consultation strategies. Gilbourne's conclusion is clearly fanciful but the basic principles behind his experiment are sound. Historical examples like this can inspire medical students to think critically about research methods and treatment strategies.

## Introduction

Basic experiments to assess the value of treatments in individual patients (i.e. rather than groups) have a long history. In 1575 the French army surgeon Ambroise Pare (1510–1590) describes applying onions (upon the advice of ‘a good old village woman’) to treat a boy scalded from falling into a ‘cauldron’ of hot oil.^1,2^ Pare observed clinical improvement in areas where scalded skin had been treated with onions. Learning from this experience, in a second patient he undertook a controlled comparison by applying onions to one-half of the patient's face. Upon the other half, he applied the ‘usual remedies’, thus making a direct comparison between the two treatments.^1,2^ Pare concluded that onions were associated with improved outcomes.

Another method for testing treatments in an individual patient lies in punctuating (‘crossing-over’) treatment with periods free from the chosen intervention. An advanced form of this method allows more than one treatment to be compared. Early examples of basic cross-over trials include that of Richard Wiseman (1622–1676), surgeon to King Charles II.^1,3^ In 1676 Mr Wiseman fitted laced stockings to a patient with dropsy (fluid retention of the legs). This was associated with improvement but later the patient desisted from wearing the stockings on the advice of another individual. When Mr Wiseman advised wearing them again, the patient improved.^1,3^

Other early cross-over studies include those of the English physician Caleb Parry (1755–1822) who in 1786 compared the purgative effects of Turkish and (cheaper) English rhubarb in individual patients.^1,4^ A further seminal study using crossed-over interventions was John Haygarth's (1740–1827) comparison in 1799 of the therapeutic benefits of Elisha Perkins' ‘metallick tractors' (alleged to possess healing properties) with (placebo) wooden 'false tractors'.^1,5^ Haygarth ensured his patients were blinded to the particular treatment intervention they were receiving.

More recently, the first modern cross-over study was led by the zoologist Lancelot Hogben (1895–1975).^1,6^ In his cross-over study, Hogben was the patient and received punctuated treatments for thyroid disease.^1,6^

**Figure 1.**
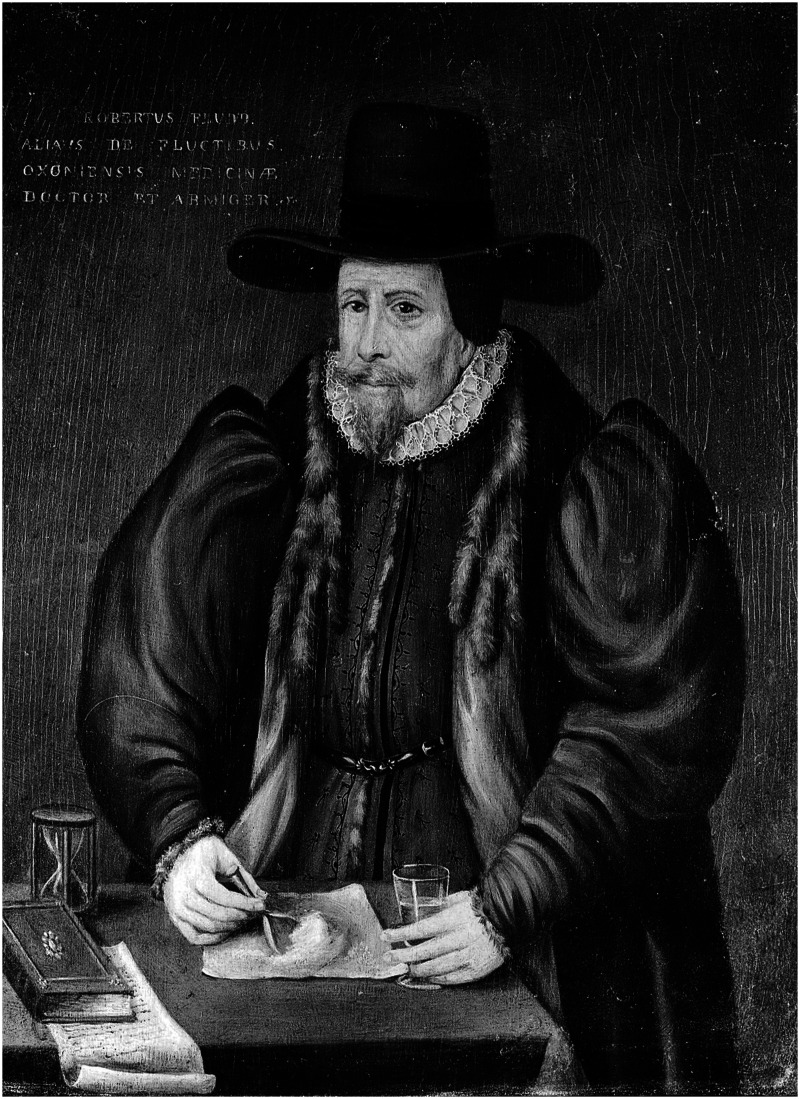
Robert Fludd (1574–1637). The hourglass is of particular note although its specific purpose (just possibly for measuring the pulse) is unknown. Image courtesy of the Wellcome Collection.

Below, we consider a cross-over trial from the seventeenth century undertaken by Sir Nicholas Gilbourne (1560–1632) and published by Robert Fludd in his 1631 book on ‘weapon salve’ (Figure 1).^7^

## Robert Fludd, Sir Nicholas Gilbourne and ‘weapon salve’

Weapon salve was a treatment (powder or ointment) whose means of cure was considered to be ‘sympathetic’, that is, by application not to a wound but to the weapon that had inflicted it.^7^ Similarly, it was believed to heal even when applied to items soaked in the patient's blood.^7^ This ‘sympathetic’ approach derived from Paracelsus^7,8^ and was believed to work thus:

The cure is based on the theory that there is a magnetic or sympathetic relationship between the weapon and the wound which allows for action over distance to occur; the wound itself needs only to be disinfected with the patient's own urine.^8^

One believer in weapon salve was Robert Fludd (Figure 1), an English physician and writer on the occult who studied at Oxford University.^8^ Fludd's biography and publication are described elsewhere but omit that his brief case description constituted a cross-over trial. The account of testing weapon salve is included in his 1631 book entitled: ‘Doctor Fludd's Answer unto M. Foster. Or the squesing of Parson Fosters Sponge, ordained by him for the wiping away of the Weapon-Salve’.^9^ The trial was undertaken by Fludd's brother in law, Sir Nicholas Gilbourne, and conveyed to Fludd in a private letter. Little is known about the life of Sir Nicholas but online sources shed some light:

Sir Nicholas Gilbourne, elder son of William Gilbourne, a businessman in London, and his wife, Alice Hunt, made his home at Charing in Kent [southeast England]… [Sir Nicholas] was appointed a Justice of the Peace for the county of Kent, sitting regularly on the bench, and was knighted by 1605. … In 1583 at her home village of Bearsted he married Joan Fludd and over the years they had ten children^10^

Sir Nicholas died around 1632 aged 72 in Charing, Kent, England.^10^ His enquiry into the effect of weapon salve is recounted by Fludd, who makes it clear that he is quoting directly from Sir Nicholas:

‘A box of this oyntment was bestowed on [Sir Nicholas]; what wholsome effects it hath wrought, I will in a word relate unto you, and this *verbatim* as I have it under his own hand’.^9^

## Sir Nicholas’ cross-over trial

Fludd introduces the trial thus:

The first [case history] (saith hee [i.e. Sir Nicholas]) was at Chatam in Kent, where the servant of one *poppee* [presumably the surname of a man called Poppy] a shipwright, was cut with his axe into the instep [i.e. sole of his foot], so deepe as it could passe, and not cut it off; upon the hurt [i.e. accident] (which was in the after noone) hee was brought unto me; but I refused to meddle with it, only I advised him, to wash his wound with his owne urine, which he did.^9^

Washing the wound in urine may have been a safer option than using water at that time. Irrigating a wound with the patient's own urine was also considered an important step in the use of weapon salve.^8^ Other than applying urine, no other treatment is mentioned. After this period without further treatment, Sir Nicholas undertakes his (magical) intervention by applying weapon salve to the axe:

The next morning early I did dresse [i.e. apply weapon salve ointment to] the axe, and after dressing it, I did send to know, how the fellow did? Answer was made that hee had beene in great paine all the night; but now lately was at ease.^9^

Sir Nicholas’ next step was to curtail his (magical) intervention and to seek the apparent consequences of this change:

The next morning comming into my study, I strucke my Rapier [i.e. a narrow straight sword] downe upon the Axe, the hilt whereof strucke the oyntment off from the axe, which when I found [i.e. that the ointment was removed], I sent to understand how hee did? and had answer that he had beene exceeding well that night; but this morning he was in great paine, and so continued [in discomfort].^9^

Finally, Sir Nicholas’ repeats the original (magical) intervention:

‘I therefore anointed the axe againe, and then sent againe unto him, and heard that hee was then at great ease: and within seven dayes was perfectly well’.^9^

Fludd appears to recognise the cross-over value of Sir Nicholas' interventions:

‘For else, why should the ointment on the axe, being discovered [i.e. removed] or struck off by the sword hilts, be an occasion of the suddaine alteration in the wound from better to worse? And why should the wound returne againe from his dolorous distemper unto his wonted ease, after the re-anointing and covering anew of the Weapon?'.^9^

## Discussion

The assumed mechanism of action of weapon salve is clearly fanciful. Nevertheless, Sir Nicholas did not consider it magical and his personal opinion was that his ointment was ‘lawfull… and no way superstitious or diabolicall’.^9^ It is likely that any clinical benefits arose from irrigation with urine (a sterile fluid) and avoidance of unnecessary surgical procedures. The latter risked the introduction of infection from unwashed instruments and grubby fingers.

Beyond extolling magic, there are obvious methodological flaws. Despite this, Sir Nicholas’ study offers two strengths: foremost is his multiple cross-overs (no treatment – treatment – no treatment – treatment). Second, is the apparent blinded nature of the patient to the (magical) treatment that he was receiving, at least by dint of his geographical remoteness. Unfortunately, neither Gilbourne or Fludd states categorically whether the patient and messenger were blinded to the intervention and to the desired positive outcome. These are important because of the placebo effect and risk of inaccurate reporting of the patient's clinical condition: that is, reporting the very results desired by a knight of the realm. Although Sir Nicholas’ original letter has not survived, it is of note that his study was documented (albeit privately) and, ultimately, published for wider dissemination.

## Conclusion

Despite invoking the power of magic, Sir Nicholas’ account offers an early illustration of a cross-over trial. To the modern eye, the fallacies in Sir Nicholas’ method are writ large in his narrative. Indeed, his account offers an early example of ‘Crabtree's bludgeon’: the tendency to make data fit a desired explanation.^11^ What is more, it suggests that in the absence of any rational explanation, a supernatural one will arise. This seems to be particularly likely wherever cause and effect are geographically remote. Under such circumstances, a further factor is likely to beget absurdity in lieu of logic: personal financial profit. The reason for such deep faith in weapon salve is unstated but we speculate that it ultimately lay in financial gain. Remote healing of injuries would have proved profitable to physicians by creating a new market for their work in surgical cases. What is more, it would have been attractive as the treatment involved no blood, guts or gore and could even be undertaken from the physician's own home. Under these circumstances, manufacture or ownership of an approved salve would likely have been particularly lucrative and convenient.

We note that historical accounts have value in teaching medical students about research methods. Historical vignettes – even a magical one ­– can inspire medical students to think critically about research methods. That is because historical studies reveal the challenges of research design in diverse, real-world contexts. The history of treatment trials also reveals incremental approaches to improving the quality of evidence. This is important because we have observed a tendency among medical students to ‘leap’ to the assumption that evidence gaps can be addressed by nothing less than a double-blind randomised controlled trial. Despite the educational benefits of promoting the history of medicine in the academic formation of future doctors, we note that the UK's General Medical Council omits the subject from its Outcomes for Graduates^12^ and Medical Licensing Content Map.^13^

Cross-over approaches form part of ‘trial of treatment’ strategies that remain relevant to modern medical practice.^1^ Historical vignettes to illustrate such an approach can help students understand practical strategies at play in consultations with patients. This is particularly true for general practice, where doctors must negotiate clinical management plans that lie outside formal guidelines.^14^ Here, ‘trial of treatment’ offers a practical step towards preventing over-prescription of drugs and forms part of a wider approach of strategic ‘well-judged clinical restraint’.^15^ Assessing the value of medical treatment for individuals in this way serves to build trust with patients, to reduce overtreatment and, ultimately, to promote the environmentally sustainable use of healthcare resources.
